# Predictors of persistent symptoms after mRNA SARS-CoV-2 vaccine-related myocarditis (myovacc registry)

**DOI:** 10.3389/fcvm.2023.1204232

**Published:** 2023-06-21

**Authors:** Daniel Schroth, Ria Garg, Xhoi Bocova, Jochen Hansmann, Markus Haass, Andrew Yan, Carlos Fernando, Binita Chacko, Anastasia Oikonomou, James White, Muhammad Mustafa Alhussein, Sorin Giusca, Andreas Ochs, Grigorios Korosoglou, Florian André, Matthias G. Friedrich, Marco Ochs

**Affiliations:** ^1^ Departments of Radiology and Cardiology, Theresien Hospital, Mannheim, Germany; ^2^ Departments of Medicine and Diagnostic Radiology, McGill University Health Centre (MUHC), Montreal, QC, Canada; ^3^ Division of Cardiology, Unity Health Toronto, St. Michael’s Hospital, Toronto, ON, Canada; ^4^ Department of Medical Imaging, Sunnybrook Health Sciences Center, Toronto, ON, Canada; ^5^ Stephenson Cardiac Imaging Centre, Foothills Medical Centre, Calgary, AB, Canada; ^6^ Department of Cardiology, GRN Hospital, Weinheim, Germany; ^7^ Department of Cardiology, Angiology and Pneumology, University Hospital Heidelberg, Heidelberg, Germany

**Keywords:** covid vaccination, myocarditis, persistent symptoms, predictors, outcome

## Abstract

**Aims:**

Epidemiological surveillance has raised safety concerns for mRNA SARS-CoV-2-vaccination-related myocarditis. We aimed to analyze epidemiological, clinical and imaging findings associated with clinical outcomes in these patients in an international multi-center registry (NCT05268458).

**Methods and results:**

Patients with clinical and CMR diagnosis of acute myocarditis within 30 days after mRNA SARS-CoV-2—vaccination were included from five centers in Canada and Germany between 05/21 and 01/22. Clinical follow-up on persistent symptoms was collected. We enrolled 59 patients (80% males, mean age 29 years) with CMR-derived mild myocarditis (hs-Troponin-T 552 [249–1,193] ng/L, CRP 28 [13–51] mg/L; LVEF 57 ± 7%, LGE 3 [2–5] segments). Most common symptoms at baseline were chest pain (92%) and dyspnea (37%). Follow-up data from 50 patients showed overall symptomatic burden improvement. However, 12/50 patients (24%, 75% females, mean age 37 years) reported persisting symptoms (median interval 228 days) of chest pain (*n* = 8/12, 67%), dyspnea (*n* = 7/12, 58%), with increasing occurrence of fatigue (*n* = 5/12, 42%) and palpitations (*n* = 2/12, 17%). These patients had initial lower CRP, lower cardiac involvement in CMR, and fewer ECG changes. Significant predictors of persisting symptoms were female sex and dyspnea at initial presentation. Initial severity of myocarditis was not associated with persisting complaints.

**Conclusion:**

A relevant proportion of patients with mRNA SARS-CoV-2-vaccination-related myocarditis report persisting complaints. While young males are usually affected, patients with persisting symptoms were predominantly females and older. The severity of the initial cardiac involvement not predicting these symptoms may suggest an extracardiac origin.

## Introduction

The corona virus disease of 2019 (COVID-19) pandemic, caused by the severe acute respiratory syndrome coronavirus-2 (SARS-CoV-2), led to the quick development and approval of multiple vaccines against the disease, some of which are based on the emerging messenger RNA (mRNA) technology.

Currently, a total of 6 vaccines in Canada and 5 in Germany are authorized for prevention of COVID-19, including 2 mRNA-based vaccines: Pfizer-BioNTech (BNT162b2) and Moderna (mRNA-1273). These vaccines were the first to receive emergency use authorization by the United States Food and Drug Administration (USFDA) (1) to reduce the risk and severity of COVID-19. As of May 21st, 2022, 67% of the world population has received at least 1 dose of an approved COVID-19 vaccine. A total of more than 30 million (85% of total population) people in Canada have received at least one dose of an approved COVID-19 vaccine as of May 13, 2022 (2); this number being more than 63 million (76% of total population) people in Germany as of May 26, 2022 (3).

Numerous studies, including case reports and epidemiological research, have suggested the development of mRNA vaccine-related myocarditis and pericarditis, particularly in younger men (4). Notably, however, the clinical trials for both mRNA vaccines have not recorded nor reported myocarditis, potentially missing this adverse event (5–8).

The Centers for Disease Control and Prevention's (CDC's) review of vaccine safety data in the Vaccine Adverse Event Reporting System (VAERS) reporting system (9), from December 2020 to August 2021 showed that out of the over 350 million vaccinated: “rates of myocarditis were highest following the second dose of an mRNA vaccine among males in the age group 12–24 years.”

These data not only support previous observations but also confirm their low incidence.

CDC vaccine safety datalink identified that in the 0–7 days post-vaccination, especially after the second dose, both vaccines were associated with an increased risk of myocarditis and pericarditis in the 18–39-year-old age group; estimated to be 22.4 excess cases per million second doses after Pfizer vaccine and 31.2 excess cases per million second doses after Moderna vaccine, suggesting higher incidence after Moderna compared to Pfizer (9).

These registries however should be interpreted with caution because they are only a passive safety signal detection system accompanied by reporting bias. This alone cannot be used to derive causality or any definite conclusions (10, 11).

A study to evaluate the safety of BNT162b2 mRNA vaccine in Israel, with approximately 900,000 vaccinated participants and unvaccinated controls, concluded that the risk of myocarditis after COVID-19 infection (risk ratio = 18.28) surpasses the risk of myocarditis after BNT162b2 vaccine (risk ratio = 2.43) (12), which was reported in the CDC's morbidity and mortality weekly report (MMWR) published on July 9th, 2021 (13). CDC also recommends that the second dose of COVID-19 vaccines should be deferred in patients who had myocarditis or pericarditis after receiving the first dose, with certain exceptions, until additional data is available (14). A consensus document supported by working groups of the European Society of Cardiology has recently been published and emphasizes the rare occurrence of post-vaccine myocarditis compared to myocarditis associated with COVID-19 (15).

In recent years, cardiac MRI (CMR) has become the primary modality for the non-invasive diagnosis of myocarditis (16). A recent recommendations paper suggested that CMR is useful in patients with suspected myocarditis or myopericarditis whenever there is uncertainty in making a diagnosis or to determine the extent of injury and inflammation (17).

We aim to analyze the risk factors associated with clinical outcomes in patients with mRNA COVID-vaccine related myocarditis, diagnosed using CMR. To our knowledge, no study has analyzed the outcomes in terms of symptom severity in these patients and correlated them with CMR findings from a multi-center cohort. Our findings will give the clinician a better understanding of the clinical course, CMR imaging findings and predictors for the outcome of mRNA-vaccine related myocarditis.

## Methods

### Study design and study population

This multicenter register study (NCT05268458) includes 59 cases of SARS-CoV-2—mRNA-vaccine related myocarditis from 3 hospitals in Canada and 2 hospitals in Germany between 05/2021 and 01/2022 (7 patients from McGill University Health Centre in Montreal, Canada; 26 patients from Foothills Medical Center in Calgary, Canada; 6 patients from Sunnybrook Health Sciences Centre in Toronto, Canada; 9 patients from University Hospital Heidelberg, Germany; and 11 patients from Theresienkrankenhaus in Mannheim, Germany). The study was approved by the ethics committee of the Landesärztekammer Baden-Württemberg, Germany (F-2021-126) as well as the institutional review boards of all participating Canadian sites. All research was performed in accordance with the relevant guidelines and regulations. Informed consent was obtained from all patients or, in the case of minors, from their parent or legal guardian.

Vaccine-related myocarditis was defined as cases of clinically suspected myocarditis with positive CMR findings within 30 days after SARS-CoV-2—mRNA—vaccination without any other plausible etiology. Data on demographics, previous medical history, previous and current symptoms as well as clinical course were gathered from the patients’ records. Laboratory results including NT-proBNP, high sensitivity troponin T, GFR, leucocytes and CRP were collected. Every patient received one follow-up phone call at least 4 weeks after hospital discharge. Follow-up data included information on persisting symptoms and New York Heart Association (NYHA) functional classification.

### Image acquisition

Every patient received a CMR as part of their routine clinical work-up in the participating study site, with the site-specific clinical MRI protocol for myocarditis. MRI studies were performed on commercial 1.5 T or 3 T scanners with a standard cardiac surface or body coil (McGill University Health Centre: Signa Premier, General Electric, USA; Foothills Medical Center: Magnetom Prisma and Skyra, Siemens Healthineers, USA; Sunnybrook Health Sciences Centre: Magnetom Vida and Sola, Siemens Healthineers, USA; University Hospital Heidelberg: Ingenia and Ingenia CX, Philips Healthcare, Best, The Netherlands; Theresienkrankenhaus Mannheim: Signa Architect, General Electric, USA and Magnetom Avanto, Siemens Healthineers, Germany). All MRI protocols included standard long-axis and short-axis stack cine sequences (steady state free precession) and Late Gadolinium Enhanced (LGE) images (phase sensitive inversion recovery) acquired 10 min after administration of intravenous contrast (0.1–0.2 mmol/kg body weight). For edema sensitive sequences either standard long-axis and short-axis stack short tau inversion recovery images (STIR) and/or native T2 maps were acquired. Imaging protocols included native T1 maps (modified Look-Locker inversion recovery) in all but 12 patients. Native T2 maps were acquired for all but 10 patients. Mapping sequences were acquired as short-axis stack of at least 3 slices. T1 and T2 maps were either automatically generated on the scanner or images were transferred to a workstation for further analysis.

### Image analysis

Image analysis was performed directly at each participating study center by an experienced reader blinded to follow-up with at least 3–5 years of experience in Cardiac MRI using certified software (cvi42, Circle Cardiovascular Imaging, Calgary, Canada). Functional data including ventricular volumes, mass and bi-ventricular ejection fraction were measured and calculated after manual contour definition. The presence of LGE was evaluated visually as well as semi-quantitatively using the 5-standard deviation method for each segment of the AHA model (18). In addition, the predominant LGE pattern was identified (subendocardial, mid-wall or subepicardial). If available, T1 and T2 maps were segmented, and average relaxation times were calculated globally and for each segment according to the AHA model.

### Statistical analysis

Statistical analysis of all provided data by the participating study centers was carried out at the main study center (Theresienkrankenhaus Mannheim, Germany) using IBM SPSS Statistics for Windows (Version 21.0. Armonk, NY: IBM Corp.). Categorical variables are presented as counts (percentages) and continuous variables as means (standard deviation) or medians [interquartile ranges (IQRs)] depending on data distribution. The Shapiro-Wilk test was used to test for normal distribution. To aggregate mapping data from multiple MRI scanners, T1 and T2 relaxation times were converted to *Z*-scores (19) using the provided local reference ranges for each scanner. *Z*-scores are multiples of standard deviations from the mean of a normally distributed population. Clinical, laboratory and imaging parameters were compared between the subgroups with and without persisting symptoms using Student's *t*-test, Mann-Whitney *U*-test, Chi-Square or Fisher's exact test where applicable. Association of clinical symptoms and extent of myocardial involvement was tested using Spearman's correlation. The relationship of clinical, laboratory and imaging parameters with symptom persistence was assessed using logistic regression analysis. Due to the retrospective and multicentric character of our study, not all data endpoints were available for every patient. For univariate and multivariate regression analysis, the median value of the respective group was used to fill in missing variables. All statistical tests were two-tailed. A *p*-value < 0.05 was considered statistically significant.

## Results

### Baseline data

#### Patient characteristics

From the five participating study centers, 59 patients were included in the study. An overview of baseline data is given in Table 1. Forty-seven patients (80%) were male (male/female ratio 3.92). Mean (±SD) age was 29 ± 13 years. Fifteen patients (25%) had a history of cardiac or pulmonary disease, i.e., asthma in 8 (14%), coronary artery disease in 5 (8%), arterial hypertension in 2 (3%), and previous myocarditis in 2 (3%) patients. In 10 patients (17%), myocarditis occurred after the first vaccination dose, of which 2 (3%) received mRNA-1273 (Moderna) and 8 (14%) received BNT162b2 (Pfizer-BioNTech). In 49 patients (83%), myocarditis occurred after the second vaccination dose, of which 15 (25%) received two doses of mRNA-1273 (Moderna) and 24 (41%) received two doses of BNT162b2 (Pfizer-BioNTech). Six patients (10%) were vaccinated with BNT162b2 (Pfizer-BioNTech) as a first dose and mRNA-1273 (Moderna) as a second dose. Four patients (7%) received other vaccine combinations.

**Table 1:** General patient characteristics.

	All patients (*n* = 59)	With pers. Symptoms (*n* = 12)	Without pers. Symptoms (*n* = 38)	*p* value*
Age, years	29 ± 13	37 ± 16	28 ± 12	0.04
Male (%)	47 (80)	3 (25)	35 (92)	<0.01
Height, cm	177 ± 9	171 ± 11	178 ± 8	0.03
Weight, kg	80 ± 18	73 ± 15	82 ± 18	0.09
BMI, kg/m^2^ [IQR]	25 [22–28]	24 [22–27]	25 [22–28]	0.38
Prev. medical history (%)				
Asthma	8 (14)	1 (8)	5 (13)	1.00
Coronary artery disease	5 (8)	0 (0)	3 (8)	1.00
Hypertension	2 (3)	0 (0)	2 (5)	1.00
NYHA before symptom onset				
NYHA I (%)	59 (100)	12 (100)	38 (100)	-
Vaccination before symptom onset				
One dose mRNA-1273 (%)	2 (3)	0 (0)	2 (5)	1.00
One dose BNT162b2 (%)	8 (14)	0 (0)	7 (18)	0.17
First and second dose mRNA-1273 (%)	15 (25)	4 (33)	11 (29)	1.00
First and second dose BNT162b2 (%)	24 (41)	7 (58)	10 (26)	0.08
First dose BNT162b2, Second dose mRNA-1273 (%)	6 (10)	0 (0)	6 (16)	0.31
Other Combinations (%)	4 (7)	1 (8)	2 (5)	1.00
Clinical presentation				
Days after vaccine [IQR]	3 [2–5]	4 [2–6]	3 [2–4]	0.31
Chest pain (%)	54 (92)	10 (83)	35 (92)	0.58
Dyspnea (%)	22 (37)	8 (67)	11 (29)	0.04
Fatigue (%)	4 (7)	2 (17)	1 (3)	0.14
Nausea (%)	4 (7)	0 (0)	4 (11)	0.56
Palpitations (%)	4 (7)	0 (0)	3 (8)	1.00
Peak NYHA^a^				
NYHA I (%)	36 (63)	4 (33)	26 (70)	0.04
NYHA II (%)	8 (14)	3 (25)	5 (14)	0.39
NYHA III (%)	5 (9)	4 (33)	1 (3)	0.01
NYHA IV (%)	8 (14)	1 (8)	5 (14)	1.00
Admission to hospital (%)	51 (86)	6 (50)	36 (95)	<0.01
Duration of hospitalization, median days [IQR]	3 [1–4]	1 [0–2]	3 [2–4]	0.01
Admission to ICU (%)	17 (29)	1 (8)	11 (29)	0.25
Duration on ICU, median days [IQR]	0 [0–1]	0 [0–0]	0 [0–1]	0.32
Laboratory tests				
Peak NT-proBNP^b^, pg/ml	258.8 [141.8–430.8]	61 [61–61]	233 [154.3–434]	0.15
Peak hs-troponin-T^c^, ng/L	552 [249–1,193]	317.5 [82.8–664.8]	371 [244.4–1,052.8]	0.42
Peak CRP^d^, mg/L	28 [12.5–50.6]	7.9 [5.4–14.2]	42.2 [15.6–58.8]	0.01
Peak Leucocytes^e^, n/nl	7.8 [6.7–9.5]	7.6 [7–8.5]	7.8 [6.8–9.2]	0.76
Discharge hs-troponint-T^f^, ng/l	197 [19.5–619]	174 [73.1–459.3]	191 [14.8–532]	0.68
Discharge CRP^g^, mg/L	7.9 [3.7–24.5]	8 [5.9–8.7]	8.7 [3.6–36.1]	0.41
Discharge Leucocytes^h^, n/nl	6.5 [5.4–7.7]	6.4 [5.7–7.2]	6.4 [5.1–7.6]	0.83
ECG findings				
ST segment changes (%)	22 (37)	1 (8)	16 (42)	0.04

Numbers are presented as number (percentage), mean ± standard deviation or median [inter quartile range].

* *p* value for group comparison between patients with and without persisting symptoms using Student's-*t*, Mann-Whitney-*U*, Chi-Square or Fisher's exact test depending on data type and distribution.

^a^ Available in 57 baseline and 49 follow up cases.

^b^ Available in 22 baseline and 18 follow up cases.

^c^ Available in 49 baseline and 40 follow up cases.

^d^ Available in 48 baseline and 39 follow up cases.

^e^ Available in 49 baseline and 40 follow up cases.

^f^ Available in 49 baseline and 40 follow up cases.

^g^ Available in 35 baseline and 26 follow up cases.

^h^ Available in 47 baseline and 38 follow up cases.

#### Clinical findings

**Figure 1:**
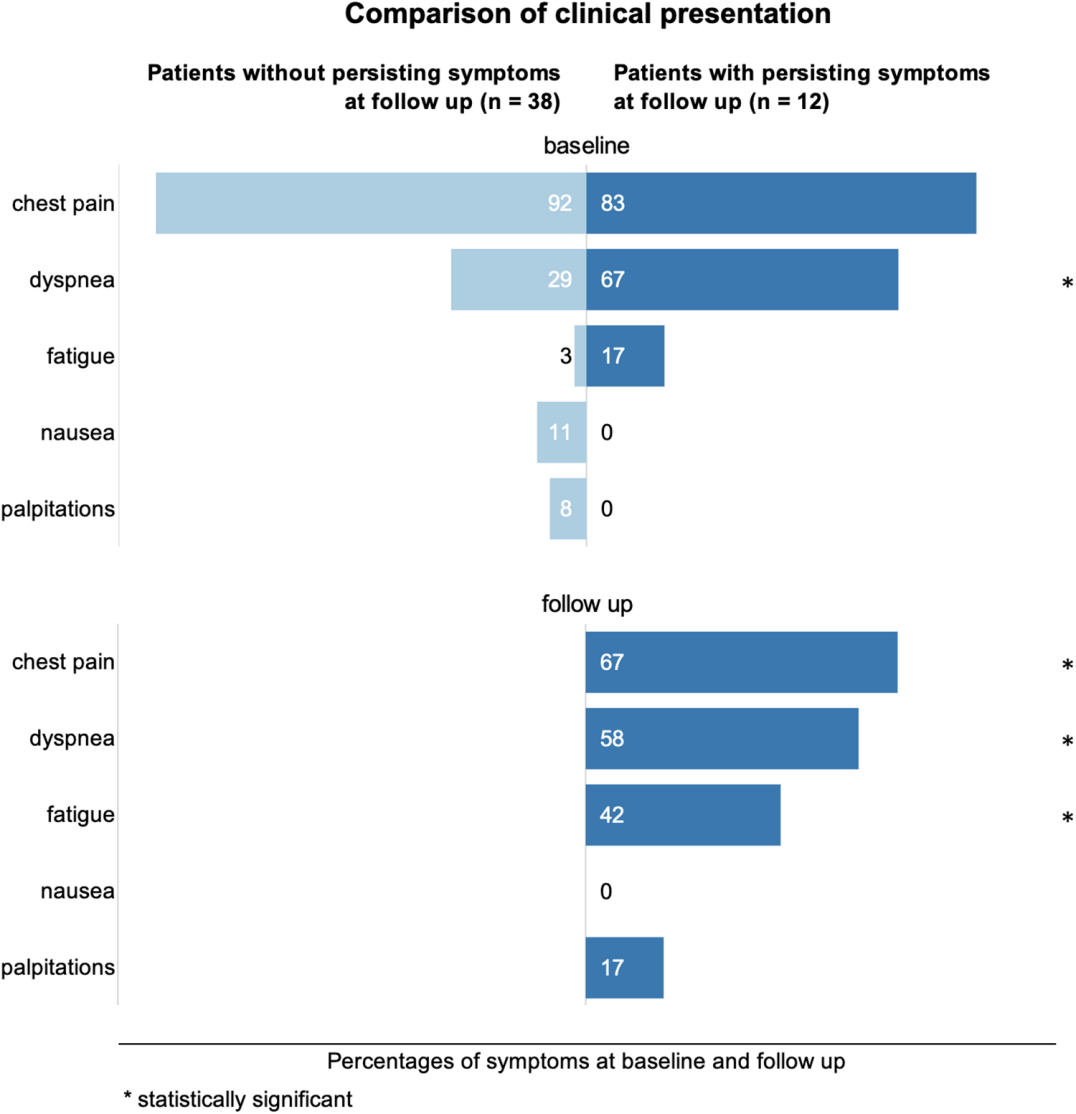
Comparison of clinical symptom occurrences in percent at baseline and follow up. Statistically significant differences between the groups with and without persisting symptoms are marked with an asterisk.

None of the patients had heart failure symptoms prior to the vaccination. The medical history of one patient included a perimyocarditis more than 10 years prior. Two patients reported a previous infection with SARS-CoV-2 without remaining symptoms prior to vaccination. Median [IQR] onset of symptoms was 3 [2–5] days after vaccination. An overview of baseline and follow up clinical symptoms is given in Figure 1. The most common symptom was chest pain, occurring in 54 (92%) of cases. Other symptoms were dyspnea in 22 (37%), fatigue in 4 (7%), nausea in 4 (7%) and palpitations in 4 (7%) of patients. 8 (14%) patients presented with dyspnea NYHA class II, 5 (9%) with NYHA class III, and 8 (14%) with NYHA class IV. 51 (86%) patients were admitted to the hospital with a median [IQR] hospitalization duration of 3 [1–4] days. 17 (29%) patients were admitted to an ICU. Twenty-two patients (37%) showed ST segment changes in the electrocardiogram (ECG). In routine laboratory workup, elevated levels of hs-troponin-T (available in 49 patients; elevated in all, 100%) and CRP (available in 48 patients; elevated in 42, 87.5%) were found. Median [IQR] peak value of hs-troponin-T was 552 [249–1,193] ng/L (reference range <14ng/L). Median [IQR] peak CRP was 28 [12.5–50.6] mg/L (reference range <5mg/L). White cell count was normal in most patients. NT-proBNP was only available in a minority of cases. All abnormal parameters improved over the course of hospital stay.

#### Imaging findings

**Figure 2:**
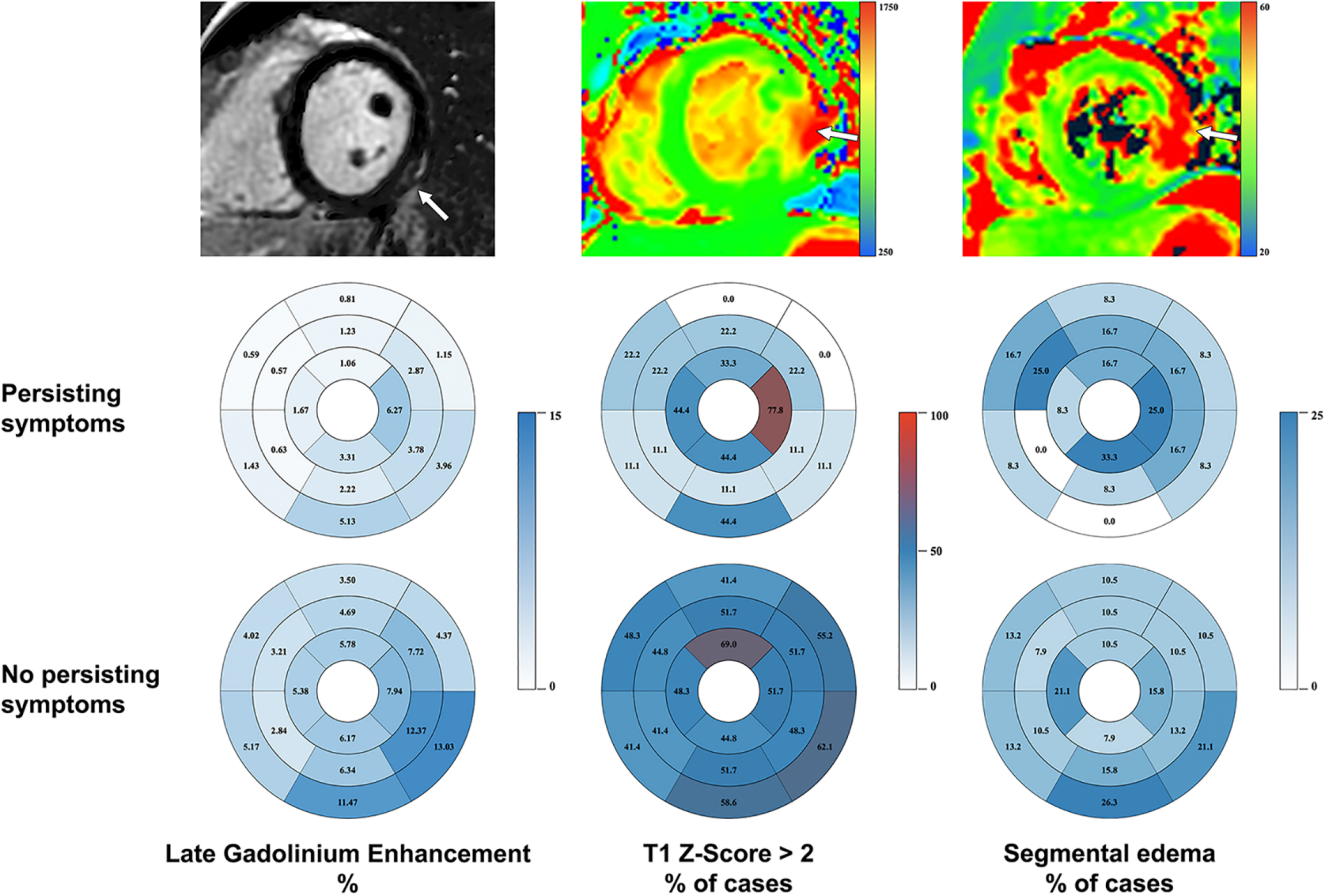
Distribution of late gadolinium enhancement, T1-mapping and edema (using STIR as fallback and T2 mapping if available) at baseline CMR. First row: typical imaging presentation of post-vaccination myocarditis in LGE, T1-mapping and fluid-sensitive sequences. A predilection for basal inferior and inferolateral segments is demonstrated (pathology marked by arrow). Second and third row: average LGE burden by segment, segmental involvement in T1-mapping and segments affected by edema for patients with and without persisting symptoms projected on the AHA model.

A summary of CMR findings is provided in Table 2. The CMR exam was performed after a median [IQR] of 3 [2–8] days after symptom onset. Most patients had a normal LV and RV systolic function with a mildly reduced left ventricular ejection fraction (LVEF <50%) in 9 (15%) cases. The dominant LGE pattern was subepicardial in 52 (91%) cases. Midwall LGE was found in 6 (11%) cases. The typical distribution of LGE as well as of T1 and T2 *Z*-scores is summarized in Figure 2, showing a predilection for the inferior and inferolateral segments at basal and midventricular planes in LGE, and a more heterogenous distribution of segments with a *Z*-score >2 in T1 and T2 mapping. However, the average segmental T2 relaxation time was relatively increased in the inferior and inferolateral basal segments and in the apex, thus, being more in line with LGE distribution. Pericardial effusion was noted in 13 (22%) and pericarditis in 7 (12%) patients.

**Table 2:** Baseline CMR results.

	All patients (*n* = 59)	With pers. Symptoms (*n* = 12)	Without pers. Symptoms (*n* = 38)	*p* value *
Left ventricle				
Ejection fraction, %	57 ± 7	62 ± 4	57 ± 7	0.03
End diastolic volume, ml	160 ± 38	128 ± 38	168 ± 37	<0.01
End systolic volume, ml	67 [55–81]	48 [43–56]	73 [62–83]	<0.01
Ejection fraction <50%, *n* (%)	9 (15)	0 (0)	7 (18)	0.17
GLS^a^, %	−15 [−17 to −13]	−14 [−16 to −13]	−15 [−17 to −14]	0.32
GCS^a^, %	−17 [−18 to −16]	−17 [−18 to −16]	−17 [−18 to −15]	0.82
GRS^a^, %	27 [25–29]	27 [26–30]	26 [23–29]	0.82
Right ventricle^b^				
Ejection fraction, %	54 ± 6	58 ± 7	53 ± 6	0.06
End diastolic volume, ml	167 [138–186]	122 [99–144]	172 [153–191]	<0.01
End systolic volume, ml	82 [57–93]	48 [42–59]	82 [62–98]	<0.01
Area of left atrium^c^, cm^2^	20 ± 4	17 ± 4	22 ± 4	<0.01
Area of right atrium^c^, cm^2^	21 ± 4	20 ± 5	20 ± 4	0.99
Pericardium				
Pericardial effusion, *n* (%)	13 (22)	0 (0)	11 (29)	0.046
Signs of pericarditis, *n* (%)	7 (12)	3 (25)	2 (5)	0.082
Late gadolinium enhancement^d^				
Subendocardial LGE, *n* (%)	0 (0)	0 (0)	0 (0)	–
Midwall LGE, *n* (%)	6 (11)	0 (0)	5 (14)	0.58
Subepicardial LGE, *n* (%)	52 (91)	7 (64)	36 (97)	0.01
Maximum segmental LGE burden^e^, %	20 [8–41]	4 [0–14]	22 [9–39]	0.01
Number of visually affected segments, median [IQR]	3 [2–5]	2 [0–4]	3 [2–5]	0.046
Native T1-mapping^f^				
Global *Z*-score, median [IQR]	1.68 [1.16–3.09]	1.2 [1.15–1.55]	2.49 [1.18–3.33]	0.04
Maximum segmental *Z*-score, median [IQR]	4.59 [3.07–6.24]	3.46 [3.17–4.03]	4.69 [3.09–6.29]	0.14
Number of affected segments (*Z*-score >2), median [IQR]	7 [2.5–11]	4 [3–5]	9 [3–13]	0.06
Myocardial edema, median [IQR] affected segments^g^	1 [0–3.5]	0.5 [0–2.5]	1 [0–2]	0.83
T2-mapping^h^				
Global *Z*-score, median [IQR]	−0.3 [−0.7 to 1]	0 [−1 to 1]	−0.3 [−0.6 to 0.5]	0.65
Maximum segmental *Z*-score, median [IQR]	2.3 [1–4]	2.4 [1–3.3]	2.1 [0.9–4.3]	0.94
Number of affected segments (*Z*-score >2), median [IQR]	1 [0–4]	1 [0–2]	1 [0–4.5]	0.80

Numbers are presented as number (percentage), mean ± standard deviation or median [inter quartile range].

* *p* value for group comparison between patients with persisting symptoms and without using Student's-*t*, Mann-Whitney-*U*, Chi-Square or Fisher's exact test depending on data type and distribution.

^a^ Available in 39 baseline and 34 follow up cases.

^b^ Available in 39 baseline and 33 follow up cases.

^c^ Available in 58 baseline and 49 follow up cases.

^d^ Available in 57 baseline and 48 follow up cases.

^e^ Available in 56 baseline and 47 follow up cases.

^f^ Available in 47 baseline and 38 follow up cases.

^g^ Using STIR as fallback and T2-mapping if available.

^h^ Available in 49 baseline and 40 follow up cases.

### Follow up

**Figure 3:**
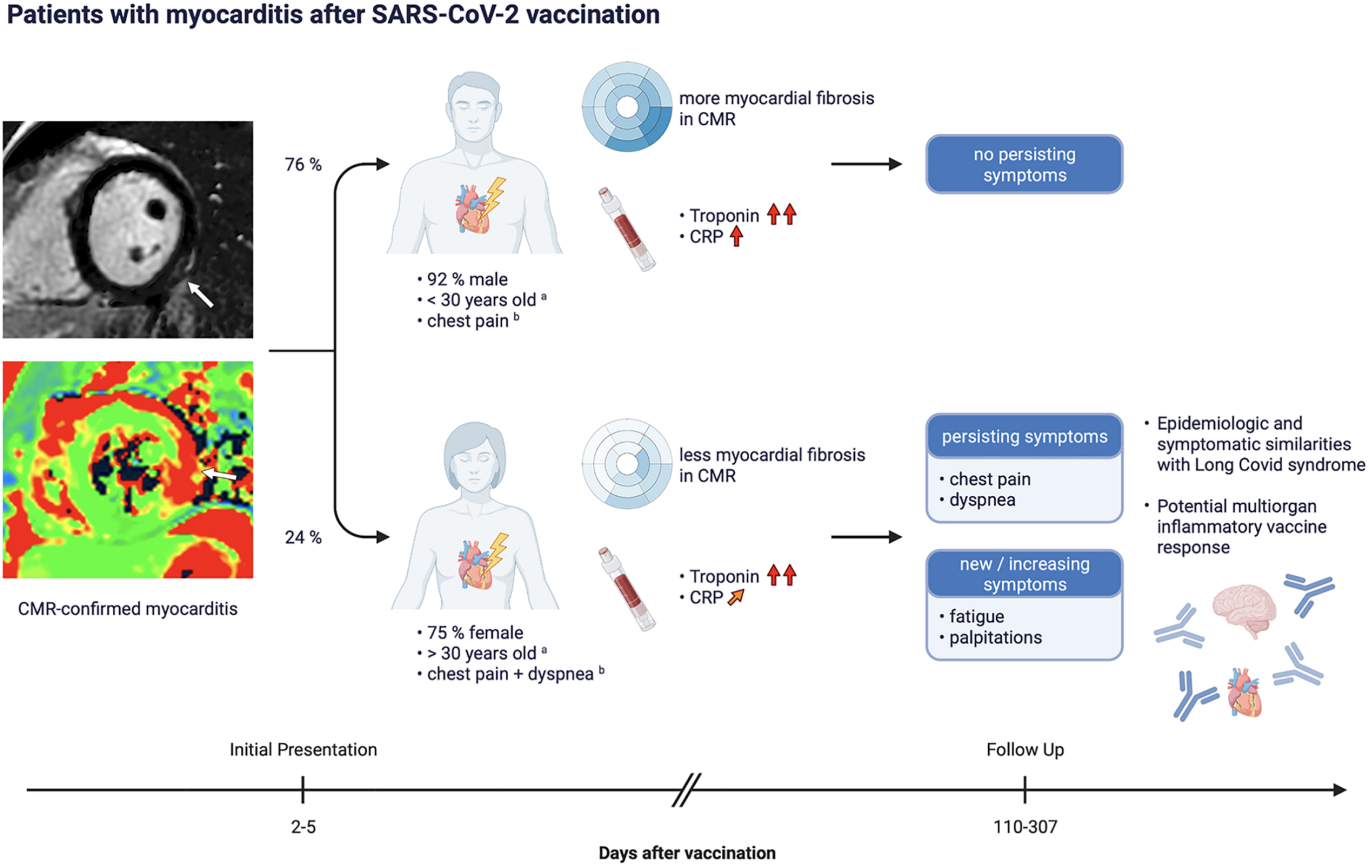
Timeline and overview of occurrence of persisting symptoms in the study population. ^a^On average, patients with persisting symptoms were females older than 30, patients without persisting symptoms were males younger than 30. ^b^Symptoms occurring in the majority of cases.

Follow-up data were available for 50 (85%) patients (9 lost to follow-up, 15%). The median [IQR] follow-up interval was 228 [110–307] days after initial diagnosis. At follow-up, 38 (76%) patients were asymptomatic while 12 (24%) patients had persisting symptoms. In the group with persisting symptoms, chest pain was reported by 8 (63%), dyspnea by 7 (58%), fatigue by 5 (42%) and palpitations by 2 (17%) patients, marking no significant reduction of chest pain and dyspnea when compared to initial presentation and even increasing rates of fatigue and palpitations. These findings are summarized in Figure 3.

#### Differences in patient characteristics and clinical presentation

As shown in Table 1, patients without persisting symptoms were predominantly male (35 men, 92%) and were 28 ± 12 years old. Patients with persisting symptoms were predominantly female (9 women, 75%) and 9 years older on average. Patients who received two doses of BNT162b2 (Pfizer-BioNTech) were significantly more likely to have persisting symptoms, while no significant difference was found for all other vaccine combinations. As can be seen in Figure 1, the spectrum of initial symptoms was different between both groups. While chest pain was the most reported initial symptom by patients with and without persisting symptoms, patients in the former group presented more often with dyspnea (8 patients, 67%) and fatigue (2 patients, 17%) as additional symptoms. In the baseline ECG, ST-segment changes occurred significantly less often in the group with persisting symptoms (1 patient, 8%). Laboratory workup revealed no difference in hs-troponin-T between both groups. However, peak CRP was significantly lower in the group of patients with persisting symptoms, showing only mildly elevated values with a median [IQR] of 7.9 [5.4–14.2] mg/L. Thirty-six patients without symptoms (95%) were admitted to the hospital at initial presentation, while only 6 patients with persisting symptoms (50%) were hospitalized.

#### Imaging findings

CMR revealed a smaller number of affected segments in LGE and native T1 mapping in the group with persisting symptoms (Table 2, Figure 2). We found no statistically significant difference between both groups in T2 mapping. The baseline left ventricular ejection fraction (LVEF) was significantly higher in patients with persisting symptoms. None of these patients displayed pericardial effusion vs. 11 (patients 29%) without persisting symptoms. However, pericardial enhancement suggestive of pericarditis was more common in the former group.

#### Predictors of outcome and extent of cardiac involvement

We used logistic regression to analyze the relationship between clinical, laboratory and imaging parameters and persisting symptoms at follow up. Table 3 summarizes the results of the univariate and multivariate regression models. Univariate analysis showed increasing odds for persisting symptoms by age, female sex, and dyspnea at initial presentation, while duration of hospitalization, peak CRP and various CMR parameters representing the severity of myocardial involvement decreased the odds. A three-parameter multivariate logistic regression model consisting of sex (female), duration of hospitalization, and peak CRP predicted persisting symptoms with an accuracy of 96% and a high level of correlation.

**Table 3:** Regression analysis for persisting symptoms in univariate and multivariate models.

Univariate analysis	Odds ratio	95% CI	*p* value
Age	1.05	1.001–1.096	0.047
Sex (Female)	35	6.022–203.433	<0.01
Dyspnea at initial presentation	5	1.223–19.709	0.03
Duration of hospitalization	0.54	0.32–0.9	0.02
Peak CRP	0.88	0.796–0.97	0.01
ST segment changes in ECG	0.13	0.015–1.069	0.06
CMR parameters			
LV Ejection fraction	1.13	1.002–1.268	0.05
LV End diastolic volume	0.97	0.943–0.99	0.01
LV End systolic volume	0.92	0.872–0.973	<0.01
RV Ejection fraction	1.12	1.003–1.247	0.04
RV End diastolic volume	0.96	0.937–0.986	<0.01
RV End systolic volume	0.91	0.862–0.968	<0.01
Area of left atrium	0.71	0.569–0.885	<0.01
Signs of pericarditis	6.00	0.869–41.443	0.07
Subepicardial LGE	0.08	0.012–0.484	0.01
Maximum segmental LGE burden	0.93	0.875–0.985	0.01
Number of affected segments in LGE	0.71	0.497–1.01	0.06
Global T1 *Z*-score	0.43	0.22–0.838	0.01
Multivariate model	Accuracy	*R*²	*p* value
	0.96	0.80	<0.01
	Odds ratio	95% CI	*p* value
Sex (Female)	67.9	2.3–1,990.5	0.01
Duration of hospitalization	0.44	0.16–1.21	0.11
Peak CRP	0.87	0.767–0.997	0.04

Values calculated using binomial logistic regression analysis for the development of persisting symptoms as dependent variable.

Correlation of symptoms at initial presentation with markers for the extent of myocardial involvement was calculated using Spearman's Rho (Table 4). A weak inverse association of dyspnea with hs-troponin-T and number of affected segments in LGE was shown. Conversely, dyspnea was positively associated with the number of affected segments in T2 mapping. Chest pain showed a weak association with number of affected segments in LGE.

**Table 4 Correlation:** of symptoms and cardiac involvement using spearman's rho.

Correlation of clinical symptoms and markers for cardiac involvement					
Peak hs-Troponin-T	Spearman's Rho	*p* value	Peak CRP	Spearman's Rho	*p* value
Chest pain	0.08	0.59	Chest pain	0.06	0.69
Dyspnea	−0.36	0.01	Dyspnea	0.00	0.97
Fatigue	0.04	0.77	Fatigue	−0.26	0.08
Nausea	−0.23	0.11	Nausea	0.03	0.85
Palpitations	−0.06	0.67	Palpitations	−0.07	0.66
LGE^a^ n of path. Segments	Spearman's Rho	*p* value	T1-Mapping^b^ n of path. segments	Spearman's Rho	*p* value
Chest Pain	0.31	0.02	Chest Pain	0.10	0.49
Dyspnea	−0.30	0.02	Dyspnea	0.05	0.74
Fatigue	−0.17	0.20	Fatigue	−0.14	0.35
Nausea	0.13	0.32	Nausea	−0.09	0.55
Palpitations	0.02	0.89	Palpitations	0.06	0.67
T2-Mapping^b^ n of path. segments	Spearman's Rho	*p* value			
Chest Pain	−0.07	0.65			
Dyspnea	0.44	0.00			
Fatigue	−0.06	0.67			
Nausea	0.03	0.83			
Palpitations	0.10	0.50			

^a^ Path. segment defined as visual presence of LGE.

^b^ Path. segment defined as segmental *Z*-score >2.

## Discussion

The aim of this multi-center study was to characterize the clinical course and outcome of vaccine-associated myocarditis after SARS-CoV-2 vaccination, evaluate the typical findings in laboratory and CMR workup as well as identify predictors for an unfavorable outcome. To our knowledge, no study has correlated clinical, laboratory and imaging parameters with patient symptoms at baseline and follow-up.

Summarizing the results from our analysis, we found that patients experiencing vaccination associated myocarditis were predominantly young males (male/female ratio 3.92; mean age 29 years). Most myocarditis cases occurred after the second dose of either mRNA vaccination (83%). Most of these cases were after BNT162b2 (Pfizer-BioNTech) (56%), likely because most administered vaccine doses in Canada (20) and Germany (21) are Pfizer-BioNTech by far. The most common symptoms at baseline were chest pain (92%) and dyspnea (37%). Laboratory work up revealed elevated troponin levels in all patients and elevated CRP in most of them (87.5%). CMR at baseline showed signs of myocarditis in accordance with revised Lake Louise Criteria (16). At follow up (median follow up interval 228 days) symptoms had resolved in most cases (76%), which is an encouraging finding. However, 24% of patients reported persisting complaints. The most common symptoms at follow up were again chest pain (67%) and dyspnea (58%), followed by notably increasing rates of fatigue (42% vs. 17% at baseline) and palpitations (17%, up from 0%) within this group. Patients with persisting symptoms were predominantly females (75%) and older. Interestingly, patients with persisting symptoms at follow-up had a significantly lower peak CRP, a lower rate of ST-segment changes in ECG, and a less myocardial involvement on CMR at initial presentation. While 95% of patients without persisting symptoms at follow-up were initially admitted to the hospital, only 50% of patients with persisting symptoms had been initially admitted to the hospital.

The new mRNA-based vaccines against SARS-CoV-2 have been developed at an unprecedented pace marking a turning point in vaccine development and a testament to the role of mRNA technology. These vaccines are generally considered safe with the most frequent side effects reported by the CDC being pain, swelling and redness at the site of injection, all of which being more pronounced after the second dose (22). Involvement of the myocardium was not shown in the COVID-19 mRNA vaccine trials which could be due to the rare incidence or the faster pre-authorization with lower number of participants, requiring the need for post-marketing surveillance (5, 6) using passive and active surveillance systems like VAERS and BEST (Biologics Effectiveness and Safety, Sentinel Initiative).

Shortly after the start of the global vaccination campaign, first reports began to appear which suggested an association between vaccination and myocardial injury. By now, the epidemiology of this vaccine-related myocarditis has been investigated by multiple large scale epidemiologic trials and its incidence is estimated to be 0.34–2.13 per 100.000 administered doses, occurring more often in younger males (23–28). The estimated catchment area for our study was about 6.5 million people. However, given the existence of multiple medical facilities in the same region as our participating sites, and our suspicion of a considerable volume of unreported cases owing to the predominantly mild course of disease, the incidence of post-vaccination myocarditis cannot be estimated with confidence for this study. The general male-dominant demographic of vaccine-related myocarditis is well represented by our baseline patient collective. Interestingly, most patients with persisting symptoms at follow-up did not fall into that demographic group by being older on average (median >30 years old) and predominantly female.

CMR findings in vaccine-related myocarditis and pericarditis have been characterized in multiple studies, case reports and case series (29–36). In summary, myocardial affection has usually been described as mild and occurring predominantly in inferior and inferolateral segments with a subepicardial distribution of LGE and edema. Similarly, in our study, CMR at baseline showed that most patients had normal LV and RV function, with LGE having a predominantly subepicardial distribution (91%), in basal and midventricular, inferior and inferolateral segments. Patients who had persisting symptoms at follow-up showed a smaller extent of myocardial involvement in CMR. LGE imaging revealed a smaller amount of myocardial fibrosis which is also reflected by significantly lower global and segmental affection in native T1 mapping. T2 mapping was not statistically different between the two groups. However, pericarditis was the only CMR abnormality that was more common in patients with persisting symptoms. The extent of irreversible injury in CMR was generally small, and the LVEF was normal (>50%) in most patients. Only a minority of patients showed a mildly reduced LVEF. Differences in EF, EDV and ESV between both groups can be explained by the fact that the group with persisting symptoms was mostly females while most patients without persisting symptoms were males, considering that the median values of the mentioned parameters were within the sex-specific normal range (37).

Similar baseline troponin levels in patients with and without persisting symptoms suggest no differences in myocardial injury (38). However, patients with a mild localized myocarditis as in our study and without a significant reduction of LVEF would generally not be expected to present with prolonged dyspnea or fatigue. When considering the smaller extent of myocardial involvement in CMR and lower inflammatory laboratory markers for patients with persisting symptoms one can draw the conclusion that myocardial affection alone does not sufficiently explain these persisting symptoms. As this is a retrospective study collecting already acquired clinical data, follow-up imaging or laboratory data was not available, and a prolonged course of acute myocarditis cannot be excluded with certainty. However, a prolonged acute myocardial inflammation in patients with persisting symptoms at follow up seems implausible given the limited extent of initial myocardial involvement.

The spectrum of reported persisting symptoms in this study—especially the increasing occurrence of fatigue and palpitations—shows similarities with Long-COVID-19 syndrome (39), which is associated with female sex and age as well (40). Similar to our group with persisting symptoms only a minority of patients with post-COVID-19 sequelae show an elevation of CRP (41). Several mechanisms have been proposed regarding long-COVID-19. Multiple studies have attributed it to a dysregulated immune response (39–42). Differences in immune system reaction between men and women could explain the female predilection for persisting symptoms we observed in our study. It has been shown that men and women have different gene expression patterns in immune cells leading to differences in pathogen response (43). Additionally, the X chromosome itself has been described to be an important factor in determining the intensity of the immune response (44, 45). In a study on humoral immune reaction to a trivalent inactivated influenza vaccine, women produced a stronger response with higher antibody levels (46). A stronger immune reaction in females in response to initial myocardial impairment after vaccination, which is suspected to be immune-mediated due to similarities between the spike protein and cardiac self-antigens like α-myosin, might more often lead to a systemic dysregulation causing persisting symptoms by affecting multiple self-antigens in different organ systems (47).

Microvascular dysfunction is another mechanism which has been proposed for cardiovascular injury in acute COVID-19 and long-COVID-19 patients (48–51). Like in other studies on microvascular dysfunction in post-COVID-19 sequelae, chest-pain has been the most common symptom at follow up in our study (51–54). Whether this presumed microvascular impairment is a direct consequence of the spike protein binding to the angiotensin-converting enzyme 2 receptor or whether this triggered a local immune response in our patients remains unclear at this time. Additionally, no immunological laboratory markers or parameters regarding microvascular impairment have been collected in our study. Future immunologic and pathophysiologic studies are needed to gather evidence whether microvascular dysfunction and/or a systemic immune reaction similar to long-COVID-19 syndrome are indeed responsible for the persisting symptoms we reported.

### Limitations

Limitations of the study include the absence of a control group, lack of follow-up imaging data and the presumptive nature of the association given no biopsy or serology were routinely obtained. Due to the retrospective nature of this study, there are some inherent limitations due to differences in technical parameters and CMR protocol for image acquisition. Furthermore, the heterogeneity of available laboratory data for our patients precluded feasible statistical comparison for NT-proBNP, a parameter not measured in most patients exhibiting mild symptoms. There is also the possibility of recall bias due to the recent media attention on this condition. Since no routine SARS-CoV-2—tests were performed between hospital discharge and gathering of follow-up data, a SARS-CoV-2 infection during this time interval cannot be ruled out for all patients. As the number and vaccine distribution of the base population of all participating hospitals is not known, no epidemiological conclusions on the vaccine associated myocarditis risk should be drawn from this study. Even in this multi-center study, the patient number is relatively low due to the rare occurrence of post-vaccination myocarditis. In this study, 9 out of 59 patients were lost to follow-up, which could potentially be attributed to their mild course of disease leading to diminished motivation for participation in the follow-up process. Additionally, there is no long-term follow-up for these patients yet available, so we cannot comment on the prognosis and long-term implications.

### Conclusion

In our observational study a relevant proportion of patients with confirmed vaccine-related myocarditis reported persisting complaints. While mRNA vaccine-related myocarditis usually affects young males, these patients with persisting symptoms were predominantly females and older. There are clinical similarities between persisting symptoms after mRNA vaccine-related myocarditis and long-COVID. Since the severity of the initial cardiac involvement was not a predictor of persisting symptoms, cardiac impairment does not sufficiently explain these symptoms. Future studies will show whether immune-triggered microvascular dysfunction or a systemic inflammatory syndrome could be a potential pathogenetic mechanism.

## Data Availability

The raw data supporting the conclusions of this article will be made available by the authors, without undue reservation.
